# Phoenixin-14 as a novel direct regulator of porcine luteal cell functions^†^

**DOI:** 10.1093/biolre/ioad138

**Published:** 2023-10-10

**Authors:** Ewa Mlyczyńska, Patrycja Kurowska, Dominika Wachowska, Małgorzata Grzesiak, Joelle Dupont, Agnieszka Rak

**Affiliations:** Laboratory of Physiology and Toxicology of Reproduction, Institute of Zoology and Biomedical Research, Jagiellonian University in Krakow, Krakow, Poland; Doctoral School of Exact and Natural Sciences, Jagiellonian University, Krakow, Poland; Laboratory of Physiology and Toxicology of Reproduction, Institute of Zoology and Biomedical Research, Jagiellonian University in Krakow, Krakow, Poland; Laboratory of Physiology and Toxicology of Reproduction, Institute of Zoology and Biomedical Research, Jagiellonian University in Krakow, Krakow, Poland; Doctoral School of Exact and Natural Sciences, Jagiellonian University, Krakow, Poland; Department of Endocrinology, Institute of Zoology and Biomedical Research, Jagiellonian University in Krakow, Krakow, Poland; National Research Institute for Agriculture, Food and the Environment, UMR85, Unité Physiologie de la Reproduction et des Comportements, Nouzilly, France; Laboratory of Physiology and Toxicology of Reproduction, Institute of Zoology and Biomedical Research, Jagiellonian University in Krakow, Krakow, Poland

**Keywords:** phoenixin, SMIM20, GPR173, corpus luteum, steroidogenesis, prostaglandins, pig

## Abstract

Phoenixin is a neuropeptide with a well-established role in the central regulation of reproductive processes; however, knowledge regarding its role in the ovary is limited. One of the main active phoenixin isoforms is phoenixin-14, which acts through G protein–coupled receptor 173. Our research hypothesis was that phoenixin-14 is expressed in porcine corpus luteum and exerts luteotropic action by affecting the endocrine function of luteal cells through G protein–coupled receptor 173 and protein kinase signaling. Luteal cells were cultured to investigate the effect of phoenixin-14 (1–1000 nM) on endocrine function. We showed that phoenixin-14 and G protein–coupled receptor 173 are produced locally in porcine corpus luteum and their levels change during the estrous cycle. We detected phoenixin-14 immunostaining in the cytoplasm and G protein–coupled receptor 173 in the cell membrane. Plasma phoenixin levels were highest during the early luteal phase. Interestingly, insulin, luteinizing hormone, progesterone, and prostaglandins decreased phoenixin-14 levels in luteal cells. Phoenixin-14 increased progesterone, estradiol, and prostaglandin E_2_ secretion, but decreased prostaglandin F_2α_, upregulated the expression of steroidogenic enzymes, and downregulated receptors for luteinizing hormone and prostaglandin. Also, phoenixin-14 increased the expression of G protein–coupled receptor 173 and the phosphorylation of extracellular signal-regulated kinase 1/2, protein kinase B, inhibited the phosphorylation of protein kinase A, and had mixed effect on AMP-activated protein kinase alpha and protein kinase C. G protein–coupled receptor 173 and extracellular signal-regulated kinase 1/2 mediated the effect of phoenixin-14 on endocrine function of luteal cells. Our results suggest that phoenixin is produced by porcine luteal cells and can be a new regulator of their function.

## Teaser

The levels of phoenixin-14 and G protein–coupled receptor 173 in the corpus luteum change during the estrous cycle in pigs and phoenixin-14 is downregulated by insulin, luteinizing hormone, P_4_, PGE_2_, and PGF_2α_; moreover, phoenixin-14 regulates steroids and prostaglandin synthesis via G protein–coupled receptor 173 and extracellular signal-regulated kinase 1/2 kinase.

## Graphical Abstract

**Graphical Abstract:**
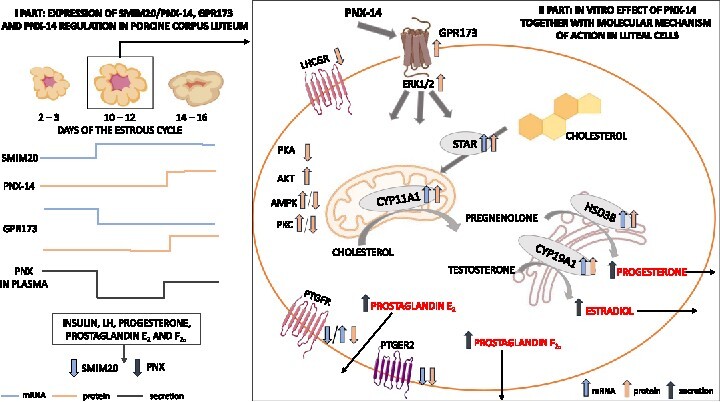

## Introduction

Neurohormonal regulation of the reproductive axis occurs primarily in the central nervous system. This regulation affects the function of gonads by controlling the secretion of gonadotropin-releasing hormone (GnRH) and gonadotropins. Recent evidence has shown that neuropeptides can be produced in the ovary and locally regulate several crucial processes such as folliculogenesis, steroidogenesis, and oocyte maturation [1]. So far, the role of kisspeptin, orexin, or ghrelin in the regulation of both pituitary and ovarian functions was well established [1–3]. In 2013, thanks to bioinformatic approach, Yosten et al. [4] identified a highly conserved neuropeptide, namely, phoenixin (PNX). Its discovery has shed new light on the central regulation of gonadotropin secretion and, as it has turned out, the entire reproductive tract. Hence, PNX is considered as a reproductive peptide.

The PNX is produced by proteolytic cleavage of the precursor small membrane integral protein 20 (SMIM20). There are many PNX isoforms of variable lengths, among which PNX-20 and PNX-14, with 20 and 14 amino acids, respectively, are predominantly produced and can cause biological effects [4, 5]. The PNX-14 is identical in humans, mice, rats, pigs, and dogs, while there is a single amino acid difference in PNX-20 between humans and pigs [4]. Most of all, high expression of this peptide has been noted in several regions of the hypothalamus such as the arcuate nucleus and the anteroventral periventricular nucleus, which are responsible for the control of reproductive processes [4], as well as other areas of the brain and spinal cord [4, 5]. The PNX is also abundantly expressed in peripheral tissues, including the heart, thymus, stomach, esophagus, liver, and kidney [4, 6]. The presence of PNX has also been documented in various ovarian structures in humans, rodents, and fish [7–10]. The expression pattern is tissue and species specific but is also related to the pleiotropic function of PNX. Almost a decade of research on the role of this peptide has provided evidence that PNX regulates food intake, improves memory, reduces anxiety, has a cardioprotective effect, regulates insulin secretion, and promotes adipogenesis [11, 12]. The PNX exerts its effect by binding to and activating G protein–coupled receptor 173 (GPR173), also called super conserved receptor expressed in brain 3 [13, 14].

The PNX is considered as a peptide that regulates the female reproductive system [15]. It is a potent stimulator of luteinizing hormone (LH) secretion from the pituitary gland of rodents and fish [4, 8, 13]. In addition, silencing the *Gpr173* gene with small interfering RNA (siRNA) reverses this effect and consequently delays estrous by 2 days [13]. In fish, PNX-20 also stimulates kisspeptin and follicle-stimulating hormone (FSH) secretion [8, 10]. Besides central regulation of reproductive processes, PNX is secreted locally in the ovary and regulates its function. In vitro experiments on human non-luteinized granulosa cell (Gc) line (HGrC1) demonstrated that PNX-20 significantly increases proliferation, estradiol (E_2_) secretion, and the number of ovulated mouse oocytes [7]. In fish, PNX similarly stimulates ovarian steroidogenesis by upregulating the expression of steroidogenic enzymes [10]. Moreover, some reproductive disorders are associated with altered levels of PNX/GPR173: the levels are higher in polycystic ovary syndrome (PCOS) [9, 16] while lower in endometriosis and cystic endometrial hyperplasia [17, 18].

Although many reports indicate that PNX is an important regulator of female reproduction, researchers have not yet attempted to determine the expression or the role of this neuropeptide in the corpus luteum (CL). The CL is a transient endocrine gland that is formed during each menstrual cycle after ovulation and regresses in the absence of fertilization of the oocyte to ensure the correct cyclicity of the ovary. It secretes large amounts of hormones, especially progesterone (P_4_), which prepares the environment for embryo implantation and subsequently maintains a pregnancy [19]. In addition, the CL secretes a number of factors like prostaglandins that can regulate its function in an autocrine manner [20]. The secretory functions of the CL are mediated by the activation of intracellular transmitters. It is known that the activation of protein kinase A (PKA) and extracellular signal-regulated kinase 1/2 (ERK1/2) leads to the phosphorylation of cAMP response element-binding protein leading in consequences to the activation of a cascade of steroidogenic factors such as steroidogenic acute regulatory protein (STAR), cytochrome P450 family 11 subfamily A member 1 (CYP11A1), hydroxy-delta-5-steroid dehydrogenase (HSD3B), and cytochrome P450 family 19 subfamily A member 1 (CYP19A1) in luteal cells [21]. What is important in the context of our study, Nguyen et al. [7] detected a strong immunohistochemical signal of PNX/GPR173 in the human CL, giving rise to their physiological importance. Thus, we hypothesized that PNX-14/GPR173 are locally expressed in CL and that PNX-14 may be luteotropic factor by stimulating the endocrine function of porcine luteal cells. Our study was designed firstly to examine the transcript and protein expression of SMIM20/PNX-14 and GPR173, and immunolocalization in porcine luteal cells during the estrous cycle and plasma PNX levels. Next, we evaluated the effect of insulin, LH, P_4_, and prostaglandin E_2_ (PGE_2_) and prostaglandin F_2α_ (PGF_2α_) on the expression and secretion of SMIM20/PNX-14 by luteal cells. We used in vitro cultures of luteal cells from days 10 to 12 of the estrous cycle to test the direct effect of PNX-14 on the luteal cell endocrine function by studying the synthesis of steroids (P_4_ and E_2_) and prostaglandins (PGE_2_ and PGF_2α_). We also examined the involvement of GPR173 and the ERK1/2 and PKA signaling pathways in the effect of PNX-14 on luteal cells.

## Materials and methods

### Ethics statement

This study used porcine tissue obtained post-mortem from a local slaughterhouse. The use of animals was in accordance with the Act of 15 January 2015 (Journal of Laws Dz. U. 2015 no. item 266) on the Protection of Animals Used for Scientific or Education Purposes and Directive 2010/63/EU of the European Parliament and the Council of the 22 September 2010 on the protection of animals used for scientific purposes, so approval from the ethics committee was not necessary.

### Reagents

Medium 199 (M199; cat. no. M2154), fetal bovine serum (FBS; cat. no. F9665), insulin (cat. no. I5523), LH (cat. no. L6420), P_4_ (cat. no. P8783), PGE_2_ (cat. no. P0409), PGF_2α_ (cat. no. P5069), Laemmli buffer (cat. no. 38733), Tris, KT5720 (product no. K3761), sodium deoxycholate, Nonidet NP-40, sodium dodecyl sulfate (SDS), protease inhibitors, dithiothreitol (DTT), Tween 20, and bromophenol blue were obtained from Sigma-Aldrich (St. Louis, MO, USA). Porcine PNX-14 amide (cat. no. 079-01) for cell culture was bought from Phoenix Pharmaceuticals Inc. (Burlingame, CA, USA). Bovine serum albumin (BSA, cat. no. ALB001) was obtained from BioShop (Ontario, Canada). The Tissue Protein Extraction Reagent (TPER; cat. no. 78510), electrophoresis markers, TaqMan Gene Expression Cells-to-CT Kit (product no. AM1728), Lipofectamine 3000 (product no. L3000001), antibiotic–antimycotic solution, and trypsin (cat. no. 26616) were purchased from Thermo Fisher Scientific (Waltham, MA, USA). PD98059 (cat. no. 1213) was obtained from Tocris (Bristol, UK).

### Sample collection and in vitro experiments

The study was carried out on cross-breed mature gilts (Large White × Polish Landrace) at the age of 7–8 months and weighing 140–150 kg. A few minutes after slaughter, the ovaries were removed, placed in phosphate-buffered saline (pH 7.4, 4 °C) with a mixture of antibiotics, and transported on ice to the laboratory within 1–1.5 h.

To determine the mRNA and protein expression of SMIM20/PNX-14 and GPR173, CL were collected from several time points of the estrous cycle: days 2–3 (early luteal phase), days 10–12 (mid-luteal phase), and days 14–16 (late luteal phase) (*n* = 6 per group, from 6 different animals) (Supplementary Figure 1). The phase of the estrous cycle was confirmed based on the morphology of ovaries [22]. Isolated CLs were frozen immediately in liquid nitrogen and stored at −70 °C for further real-time polymerase chain reaction (PCR) and western blot analysis. To examine the cellular distribution pattern of PNX-14 and GPR173, ovaries were collected at days 2–3, 10–12, and 14–16 of the estrous cycle (*n* = 4 per group, from 4 different animals) and immersed in 4% buffered formalin for immunohistochemistry. In addition, blood was collected, centrifuged at 2000*g* for 10 min at 4 °C, and stored at −20 °C for future determination of plasma PNX levels during the estrous cycle.

For in vitro experiments, porcine CL from days 10 to 12 of the estrous cycle were collected (*n* = 6 per group, six independent in vitro cultures of luteal cells). Luteal cells were isolated and prepared according to Gregoraszczuk [23]. After isolation, luteal cells were resuspended in M199 with 10% FBS [v/v]. The viability of the cells checked with the trypan blue exclusion test was 85–90%. The cells were subsequently plated in 96-well tissue culture plates at a concentration of 6 × 10^4^ cells per well for 24 h. After preincubation, the medium was replaced with M199 containing 1% FBS [v/v] and cells were treated with appropriate hormones. All cultures were maintained at 37 °C in a humidified atmosphere of 5% CO_2_ and 95% O_2_. The following experiments were performed to investigate the effect of PNX-14 on CL function.

Experiment 1: to determine the direct effect of hormones on *SMIM20* mRNA levels as well as PNX-14 protein secretion in culture medium, cells were treated with insulin at 10 ng/mL; or LH at 50, 100, or 150 ng/mL; or P_4_ at 10, 100, or 1000 nM; or PGE_2_ and PGF_2α_ at 10, 250, or 500 ng/mL for 24 h. These doses were chosen based on previous studies. Doses of hormones used in experiments were established based on our previous studies [24, 25]. Doses of LH, P_4_, PGE_2_, and PGF_2α_ correspond to the dynamic changing concentrations of these hormones during the porcine estrous cycle. In the case of insulin, one mean physiological dose (10 ng/mL) observed during the estrous cycle was selected [24, 25]. Following incubation, culture medium was harvested and then centrifuged at 1000*g* for 10 min at 4 °C. Supernatants were stored at −20 °C to analyze the PNX concentration, while cells were stored at −70 °C for further isolation of RNA to analyze *SMIM20* expression.

Experiment 2: to study the effect of PNX-14 on steroidogenesis, cells were treated with PNX-14 at 1, 10, 100, or 1000 nM and 100 ng/mL of LH alone or with 10 nM of PNX-14. For steroidogenesis, the effect of PNX-14 (10 nM) induced by LH was studied; LH is a well-known stimulator of steroid synthesis in luteal cells [26]. The PNX-14 doses were chosen based on previous studies concerning the effect of PNX on ovarian physiology [7, 10] and our preliminary data. The plasma level of PNX during the estrous cycle obtained in our study fluctuated around 1 ng/mL which corresponds to 7 nM of PNX and is included in the range of doses used in in vitro study. In addition, the dose of 10 nM of PNX-14, which is the closest to the concentration obtained in plasma, was selected for stimulation with LH and the study of the mechanism of PNX-14 action in luteal cells. To evaluate prostaglandin secretion and expression of their receptors (PTGER2 and PTGFR), cells were treated with 1–1000 nM PNX-14. Following incubation, culture medium was harvested and centrifuged at 1000*g* for 10 min at 4 °C. The supernatants were stored at −20 °C to analyze P_4_, E_2_, PGE_2_, and PGF_2α_ levels, while the cells were stored at −70 °C to analyze STAR, CYP11A1, HSD3B, CYP19A1, PTGER2, and PTGFR mRNA and protein levels as well as protein expression of LH receptor (LHCGR).

Experiment 3: to investigate the effect of PNX-14 on the activation of protein kinases, luteal cells were treated with 10 nM PNX-14 for 1, 5, 15, 30, 45, and 60 min. After treatment, the cells were frozen at −20 °C to check the protein expression of the following phosphorylated and total form of kinases: ERK1/2, PKA, protein kinase B (AKT), AMP-activated protein kinase alpha (AMPKα), and protein kinase C (PKC). In addition, the effect of PNX-14 (1–1000 nM) on GPR173 protein expression was determined.

Experiment 4: finally, the molecular mechanism by which PNX-14 affects the endocrine function of porcine luteal cells was examined. After preincubation and starvation, luteal cells were pre-treated with GPR173 siRNA (2 nM) for 24 h or pre-treated for 1 h with the ERK1/2 inhibitor PD09859 (50 μM) or the PKA inhibitor KT5720 (50 ng/mL). Finally, 10 nM PNX-14 was added for 24 h. The culture medium was collected and stored at −20 °C to measure the P_4_, E_2_, PGE_2_, and PGF_2α_ levels.

### RNA isolation and real-time PCR

Total RNA was extracted from whole CLs using the Trizol reagent (Sigma-Aldrich, Saint-Quentin-Fallavier, France), following the manufacturer’s procedure as described previously [27]. The quantity and quality of the isolated RNA were determined spectrophotometrically. Next, 1 μg of RNA was reverse transcribed for 1 h at 37 °C in a reaction containing 50 mM Tris–HCl (pH 8.3), 75 mM KCl, 3 mM MgCl_2_, 200 μM of each deoxynucleotide triphosphate (Amersham, Piscataway, NJ, USA), 50 pmol of the oligo(dT)15 primer, 5 U of ribonuclease inhibitor, and 15 U of MMLV reverse transcriptase. Next, cDNA was diluted 1:5. Real-time PCR was performed in a final volume of 20 μL containing 10 μL of iQ SYBR Green Supermix (Bio-Rad, Hercules, CA, USA), 0.25 μL of each primer (10 μM), 4.5 μL of water, and 5.0 μL of template. The MYIQ Cycler real-time PCR system (Bio-Rad) was used following the protocol previously described by Rak et al. [28]. For normalization, cyclophilin A (*PPIA*) and ribosomal protein L19 (*RPL19*) served as housekeeping genes. The primer sequences are as follows: PPIA (forward 5′-GCATACAGGTCCTGGCATCT-3′ and reverse 5′-TGTCCACATGCAGCAATGGT-3′), RPL19 (forward 5′-ACCGCCACATGTATCACAGTC-3′ and reverse 5′-TGTGCTCCATGAGAATCCGC-3′), SMIM20 (forward 5′-CTACTTCCGGCCCCTAATGC-3′ and reverse 5′-GCCCCTCAGGATTCAGCAAG-3′), and GPR173 (forward 5′-TGTTTGTGAAAGCCTGCGCC-3′ and reverse 5′-TGTTGAGCAGGAAGCAGACG-3′). The specificity of the amplified fragment sequence was assessed by Beckman Coulter Genomics (Brea, CA, USA). The efficiency was between 1.8 and 2.0.

### TaqMan real-time PCR

TaqMan gene expression assays (Applied Biosystems, Carlsbad, CA, USA) were used to quantify mRNA expression of proteins involved in steroid synthesis as well as prostaglandin receptors [27]. Total RNA isolation and cDNA synthesis were performed following the manufacturer’s protocol for the TaqManGene Expression Cells-to-CT Kit; RNA and cDNA quantities were determined based on the optical density at 260 and 280 nm. Amplifications were performed using the StepOnePlus system (Applied Biosystems) following the manufacturer’s instructions and the TaqMan-specific primers: STAR (assay ID: Ss03381250_u1), CYP11A1 (assay ID: Ss03384849_u1), HSD3B (assay ID: Ss03391752_m1), CYP19A1 (assay ID: Ss03384876_u1), PTGER2 (assay ID: Ss03374177_g1), and PTGFR (assay ID: Ss03393819_s1). The final reaction volume of 20 μL included the TaqMan Gene Expression Master Mix and 50 ng cDNA. Relative gene expression was normalized against the reference gene PPIA (assay ID: Ss03394782_g1). Relative gene expression levels were determined according to Livak and Schmittgen [29], using the comparative cycle threshold (2^−ΔΔCt^) method.

### Western blot

Tissue preparation and western blot were performed according to published protocols [30] with some modifications. In brief, porcine CL were homogenized in 200 μL of TPER. For in vitro experiments, cells were collected and Laemmli buffer was added. Then, the samples were boiled at 95 °C for 5 min. Equal amounts of the lysate (30 μg protein per sample) were separated in SDS-polyacrylamide gels and transferred onto polyvinylidene fluoride membranes. The membranes were incubated for 1 h in 0.02 M Tris-buffered saline containing 5% BSA and 0.1% Tween 20, and then incubated overnight at 4 °C with the primary antibody. Table 1 provides the details for the primary antibodies used in the study. Subsequently, membranes were washed with Tris-buffered saline containing 0.1% Tween 20 (TBST) and incubated for 1 h at room temperature (RT) with horseradish peroxidase (HRP)-conjugated antibody diluted at 1:1000 (Table 1). Signals were visualized by chemiluminescence using the WesternBright Sirius HRP substrate and the Chemidoc XRS+ System (Bio-Rad). The loading control was actin. Densitometric analysis was performed with ImageJ (National Institutes of Health, Bethesda, MD, USA).

**Table 1:** Specifications of antibodies used for western blot (WB) and immunohistochemistry (IHC) analysis

Antibody	Host	Dilution	Cat. no.	Supplier
Phoenixin-14	Rabbit	1:200 (WB)	G-079-01	Phoenix Pharmaceuticals
Phoenixin-14	Rabbit	1:100 (IHC)	H-079-01	Phoenix Pharmaceuticals
GPR173	Rabbit	1:500 (WB) 1:100 (IHC)	PA5-50976	Thermo Fisher Scientific
STAR	Rabbit	1:500	ab233427	Abcam
CYP11A1	Rabbit	1:1000	ab175408	Abcam
HSD3B	Mouse	1:1000	ab75710	Abcam
CYP19A1	Rabbit	1:200	PA1-21398	Thermo Fisher Scientific
LHCGR	Rabbit	1:200	19968-1-AP	ProteinTech
PTGER2	Rabbit	1:200	PA5-91872	Thermo Fisher Scientific
PTGFR	Rabbit	1:500	PA5-70674	Thermo Fisher Scientific
pERK1/2	Rabbit	1:1000	#9101S	Cell Signaling Technology
ERK1/2	Rabbit	1:0000	#9102S	Cell Signaling Technology
pPKA	Rabbit	1:500	ab5815	Abcam
PKA	Rabbit	1:500	ab187515	Abcam
pAKT	Rabbit	1:500	#9271S	Cell Signaling Technology
AKT	Rabbit	1:500	#9272S	Cell Signaling Technology
pAMPK	Rabbit	1:500	PA5-17831	Thermo Fisher Scientific
AMPK	Rabbit	1:500	PA5-17398	Thermo Fisher Scientific
pPKC	Rabbit	1:700	ab180848	Abcam
PKC	Rabbit	1:700	ab32376	Abcam
Actin	Mouse	1:1000	A5316	Sigma-Aldrich
Anti-rabbit	Goat	1:1000	#7074	Cell Signaling Technology
Anti-mouse	Horse	1:1000	#7076	Cell Signaling Technology

### Immunohistochemistry

Paraplast-embedded CL sections (5 μm) were processed by immunohistochemistry. Nonspecific binding sites were blocked with 5% normal goat serum for 30 min. Then, the sections were incubated with the primary antibody anti-PNX-14 or anti-GPR173 overnight (Table 1). Next, the antigens were visualized using biotinylated secondary antibodies (1300, 1.5 h at RT; Vector Laboratories, Newark, CA, USA), avidin–biotin–peroxidase complex (40 min at RT, Vectastain Elite ABC Reagent; Vector Laboratories), and 3,3′-diaminobenzidine (DAB; Sigma-Aldrich) as a chromogenic substrate. Sections were then counterstained with hematoxylin, dehydrated, and mounted in DPX (Sigma-Aldrich). For the negative control, sections were incubated with non-immune rabbit IgG (NI01; Calbiochem, Darmstadt, Germany) instead of primary antibodies and processed as described above. Selected sections were photographed using a Nikon Eclipse Ni-U microscope and a Nikon Digital DS-Fi1-U3 camera (Nikon, Tokyo, Japan) with the corresponding software.

### Enzyme-linked immunosorbent assay

The plasma and culture medium PNX levels were determined using a commercially available enzyme-linked immunosorbent assay (ELISA) kit. The manufacturer states that the assay is aimed at detecting PNX-14 but shows 100% cross-reactivity with PNX-20. Culture medium P_4_ and E_2_, PGE_2_, and PGF_2α_ concentrations were also measured with ELISA kits. Table 2 provides detailed information about the ELISA kits used in this study. Absorbance values were measured at 450 nm using a Varioskan LUX Multimode Microplate Reader and SkanIt Software 6.1.1 (Thermo Fisher Scientific).

**Table 2:** Characteristic of ELISAs used in study

Target protein	Cat. no. and supplier	Standard curve range	Sensitivity of the assay	Intra-assay coefficient of variation	Inter-assay coefficient of variation
Phoenixin	EK-079-01, Phoenix Pharmaceuticals	0.36–26 ng/mL	0.07 ng/mL	<10%	<15%
Progesterone	EIA-1561, DRG Instruments GmbH	0.14–40 ng/mL	0.045 ng/mL	<8%	<10%
Estradiol	EIA-2693, DRG Instruments GmbH	10.6–2000 pg/mL	10.6 pg/mL	<10%	<15%
Prostaglandin E_2_	EP0205, FineTest	31.25–2000 pg/mL	18.75 pg/mL	<8%	<10%
Prostaglandin F_2α_	EP0206, FineTest	7.81–500 pg/mL	4.68 pg/mL	<8%	<10%

### Gene silencing

The GPR173 silencing experiment was designed according to Nguyen et al. [7]. Luteal cells were transfected with either GPR173 or control siRNA (in a volume of 0.2 μL in 200 μL of medium per well, yielding 2 nM of siRNA) using Lipofectamine 3000 according to the manufacturer’s instructions. The sequences of porcine negative control siRNA used were as follows: UUC UCC GAA CGU GUC ACG UTT (sense) and ACG UGA CAC GUU CG AGA ATT (antisense); GPR173a (siRNA1) GAUGAAGCCAGUGCAGAUG (sense) and AAGAUGAAGCCAGUGCAGAUGGU (antisense); GPR173b (siRNA2) ACUGGACAUUCCAUGGUCC (sense) and GAACUGGACAUUCCAUGGUCCCG (antisense); GPR173c (siRNA3), GGA UGU UUA GGA GGU AUU GGC UUA A (sense) and UUA AGC CCA AUA UUU CCU AAA CAU CCA A (antisense); GPR173d (siRNA4) GGCUCCUUACUACUUCCUG (sense) and AAGGCUCCUUACUACUUCCUGCU (antisense).

### Statistical analysis

GraphPad Prism version 8.0.1 (GraphPad Software, San Diego, CA, USA) was used for all statistical analyses. The data are presented as mean ± standard error of the mean (SEM) of six replicates. All data were tested for the assumptions of normality (the Shapiro–Wilk test) and homogeneity of variances (Levene test). One-way analysis of variance was performed followed by the Tukey test. Statistical significance is indicated by different letters (*P* < 0.05) or asterisks (^*^P < 0.05, ^*^^*^P < 0.01 and ^*^^*^^*^P < 0.001, ^*^^*^^*^^*^P < 0.0001).

## Results

### Expression of SMIM20/PNX-14 and GPR173 in porcine CL and plasma PNX levels during the estrous cycle

**Figure 1:**
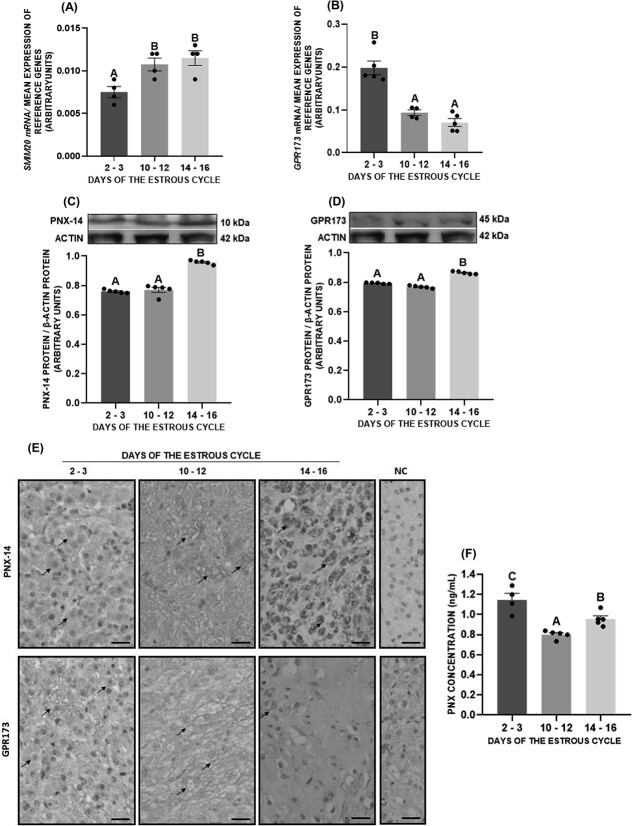
Relative mRNA and protein expression of SMIM20/phoenixin-14 (PNX-14) (A, C) and GPR173 (B, D) in porcine corpus luteum. The localization of PNX-14 and GPR173 in the porcine corpus luteum (E) as well as PNX plasma concentration was determined (F) during the estrous cycle. Black arrows point to the signal for PNX-14 and GPR173 in large luteal cells, bar = 50 μm. The data are presented as the mean ± SEM (*n* = 6). Statistical analysis was carried out using one-way ANOVA, followed by Tukey test. Different capital letters indicate significant differences at *P* <0.05. NC—negative control.

*SMIM20* mRNA levels increased in CL throughout the luteal phase, with the highest expression on days 10–12 and 14–16 of the estrous cycle, while *GPR173* expression decreased and was the lowest on those days of the estrous cycle (Figure 1A and B, *P* < 0.05). At the protein level, we observed that the expression of both PNX-14 and GPR173 expression was higher in CL on days 14–16 of the estrous cycle compared with days 2–3 and 10–12 (Figure 1C and D, *P* < 0.05). We detected PNX-14 and GPR173 immunostaining mainly in large luteal cells during the entire luteal phase, with an especially strong signal for PNX-14 on days 14–16 of the estrous cycle (Figure 1E). We noted the presence of PNX-14 in the cytoplasm and GPR173 in the cell membrane of luteal cells.

Plasma PNX levels were highest on days 2–3 of the estrous cycle (1.13 ± 0.07 ng/mL), then decreased during the mid-luteal phase (0.8 ± 0.02 ng/mL) and increased slightly on days 14–16 of the estrous cycle (0.96 ± 0.04 ng/mL) (Figure 1F, *P* < 0.05).

### The effect of insulin, LH, P_4_, PGE_2_, and PGF_2α_ on SMIM20/PNX-14 levels in porcine luteal cells

**Figure 2:**
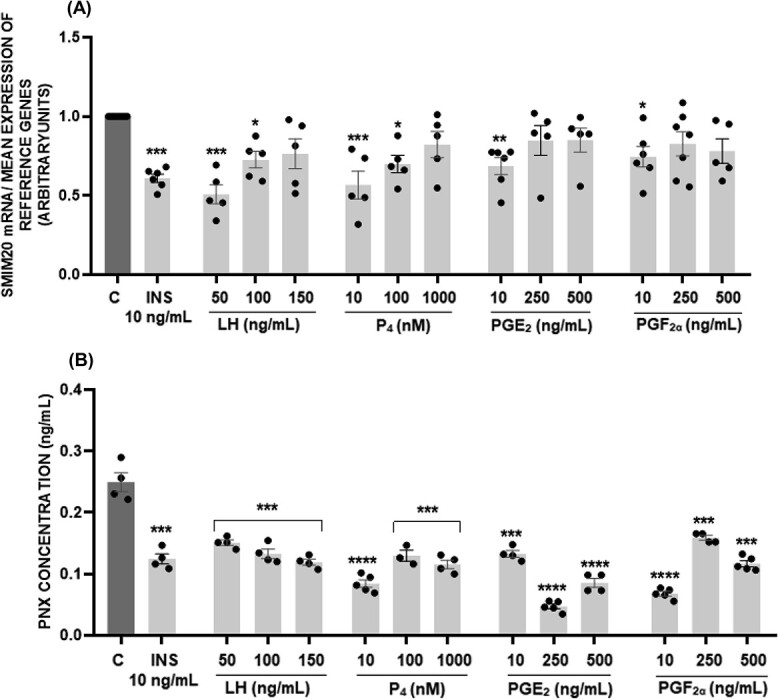
In vitro effect of insulin, luteinizing hormone (LH), progesterone (P_4_), prostaglandin E_2_ (PGE_2_), and F_2α_ (PGF_2α_) on mRNA expression of small membrane protein 20 (SMIM20) (A) and secretion of phoenixin (PNX) (B) by porcine luteal cells. The data are presented as the mean ± SEM (*n* = 6). Statistical analysis was carried out using one-way ANOVA, followed by Tukey test. Statistically significant changes compared to control were demonstrated at ^*^P < 0.05, ^*^^*^P < 0.01, ^*^^*^^*^P < 0.001, and ^*^^*^^*^^*^P < 0.0001.

We next investigated possible hormonal regulation of PNX levels in luteal cells. Insulin (10 ng/mL) and other hormones—50 and 100 ng/mL of LH, 10 and 100 nM of P_4_, and 10 ng/mL of PGE_2_ and PGF_2α_—led to a reduction in *SMIM20* mRNA levels (Figure 2A, *P* < 0.05). On the other hand, the PNX levels in the culture media indicated that all factors at the tested doses decreased PNX secretion by luteal cells compared with the control (Figure 2B, *P* < 0.001, *P* < 0.0001).

### The in vitro effect of PNX-14 on the steroidogenesis of porcine luteal cells

**Figure 3:**
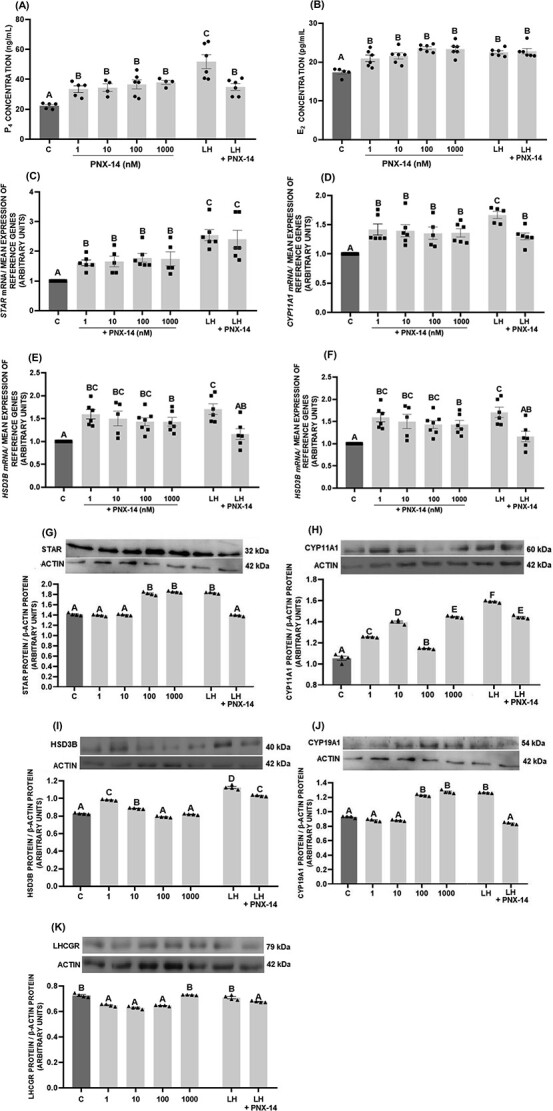
In vitro effect of phoenixin-14 (PNX-14) at doses 1–1000 nM and combined with luteinizing hormone (LH) at a dose of 100 ng/mL on progesterone (P_4_) (A) and estradiol (E_2_) (B) secretion as well as mRNA (C–F) and protein expression (G–J) of steroidogenic proteins and protein expression of LH receptor (LHCGR) (K) in porcine luteal cells. The protein abundance was analyzed by western blot method. Results are shown as representative immunoblots and a bar chart with densitometry measurement of relative target protein content normalized with actin protein. The data are presented as the mean ± SEM (*n* = 6). Statistical analysis was carried out using one-way ANOVA, followed by Tukey test. Different capital letters indicate significant differences at *P* <0.05.

PNX-14 at 1–1000 nM elevated the secretion of both P_4_ and E_2_ by luteal cells compared with the control (Figure 3A and B, *P* < 0.05). Administration of LH, as expected, also increased the secretion of P_4_ and E_2_. In contrast, PNX-14 in combination with LH reduced P_4_ secretion compared with LH alone but had no effect on E_2_.

PNX-14 increased *STAR*, *CYP11A1*, *HSD3B*, and *CYP19A1* mRNA levels while PNX-14 combined with LH decreased *CYP11A1* and *HSD3B* mRNA levels (Figure 3C–F, *P* < 0.05). Similarly, PNX-14 had a dose-dependent stimulatory effect on the protein expression of steroidogenic enzymes: 1 and 10 nM PNX-14 increased HSD3B expression, 100 and 1000 nM PNX-14 increased STAR and CYP19A1 expression, while all PNX-14 doses increased CYP11A1 expression (Figure 3G–J, *P* < 0.05). The combined administration of PNX-14 with LH reduced the expression of all the tested proteins (Figure 3G–J, *P* < 0.05). In contrast, PNX-14 at doses 1–100 nM led to downregulation of the LHCGR protein expression. We did not observe changes in LHCGR expression after LH treatment, while together with PNX-14 there was a decrease in LHCGR level (Figure 3K, *P* < 0.05).

### The in vitro effect of PNX-14 on prostaglandin secretion and PTGER2 and PTGFR expression in porcine luteal cells

**Figure 4:**
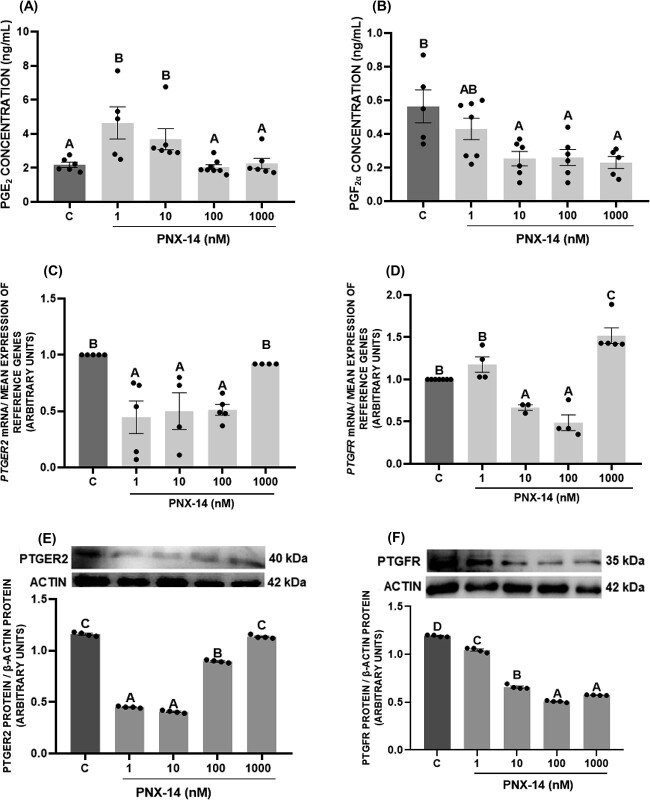
In vitro effect of phoenixin-14 (PNX-14) at doses 1–1000 nM on prostaglandin E_2_ (PGE_2_) (A) and F_2α_ (PGF_2α_) (B) secretion as well as mRNA and protein expression of PTGER2 (C, E) and PTGFR (D, F) in porcine luteal cells. The protein abundance was analyzed by western blot method. Results are shown as representative immunoblots and a bar graph with densitometry measurement of relative target protein content normalized with actin protein. The data are presented as the mean ± SEM (*n* = 6). Statistical analysis was carried out using one-way ANOVA, followed by Tukey’s test. Different capital letters indicate significant differences at *P* <0.05.

Both 1 and 10 nM of PNX-14 increased PGE_2_ levels, while 10–1000 nM PNX-14 decreased PGF_2α_ secretion (Figure 4A and B, *P* < 0.05). We also evaluated the effect of PNX-14 on the expression of prostaglandin receptors. At the mRNA and protein levels, PNX-14 at 1–100 nM downregulated the expression of the PTGER2 (Figure 4C and E, *P* < 0.05). In the case of PTGFR receptor, PNX-14 at 1–1000 nM decreased protein levels of PTGFR but had a variable effect on the mRNA levels: 10 and 100 nM reduced its expression, while 1000 nM significantly increased its expression (Figure 4D and F, *P* < 0.05).

### The in vitro effect of PNX-14 on GPR173 expression and phosphorylation of kinases in porcine luteal cells

**Figure 5:**
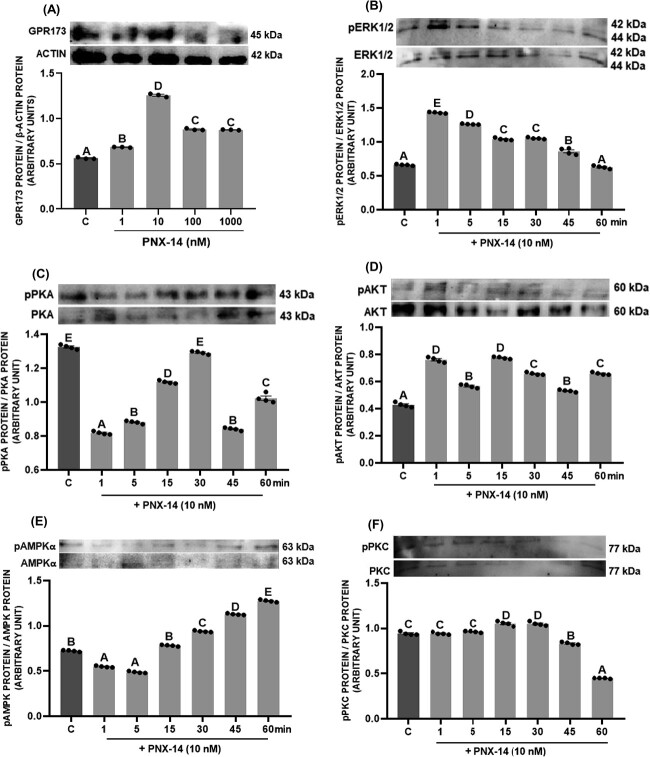
In vitro effect of phoenixin-14 (PNX-14) at doses 1–1000 nM on GPR173 protein expression (A) in porcine luteal cells and time-dependent effect of PNX-14 at doses 10 nM on activation of kinases (B–F). The protein abundance was analyzed by western blot method. Results are shown as representative immunoblots and a bar chart with densitometry measurement of relative target protein content normalized with actin protein. The data are presented as the mean ± SEM (*n* = 6). Statistical analysis was carried out using one-way ANOVA, followed by Tukey test. Different capital letters indicate significant differences at *P* <0.05.

The PNX-14 at 1 and 10 ng/mL elevated GPR173 protein levels in luteal cells (Figure 5A, *P* < 0.05). We observed that PNX-14 activated various protein kinase pathways in porcine luteal cells: depending on the time of incubation, it increased ERK1/2 and AKT phosphorylation and decreased PKA (Figure 5B–D, *P* < 0.05). In the case of AMPKα and PKC, PNX-14 has mixed effect on them activation. AKT kinase phosphorylation is initially reduced, but 30, 45, and 60 min after administration of PNX-14, there was increased AMPKα phosphorylation (Figure 5E, *P* < 0.05). In contrast, PKC phosphorylation is upregulated by PNX-14 after 15 and 30 min and then significantly downregulated in 45 and 60 min (Figure 5F, *P* < 0.05).

### The involvement of GPR173 and kinases signaling pathways in the effect of PNX-14 on steroid and prostaglandin secretion by porcine luteal cells

**Figure 6:**
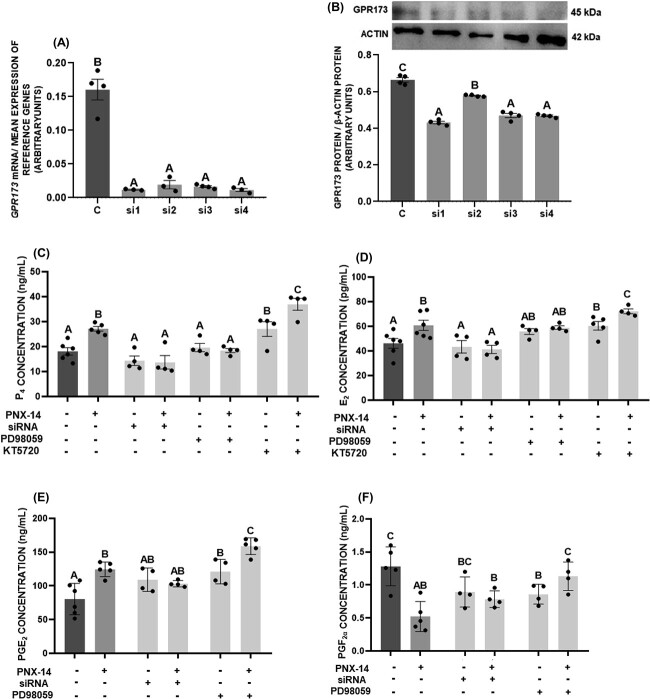
The involvement of GPR173 and extracellular signal-regulated kinases (ERK1/2) and protein kinase A (PKA) in the effect of phoenixin-14 (PNX-14) on progesterone (P_4_) (C), estradiol (E_2_) (D), prostaglandin E_2_ (PGE_2_) (E), and F_2α_ (PGF_2α_) (F) secretion by luteal cells. GRP173 gene was silenced using four different sequences of siRNA (si1-si4) and the mRNA (A) and protein (B) expression of GPR173 were analyzed to confirm its knockout in luteal cells. To block ERK1/2 and PKA kinases, pharmacological blockers PD98059 and KT5720, respectively, were used. The data are presented as the mean ± SEM (*n* = 6). Statistical analysis was carried out using one-way ANOVA, followed by Tukey test. Different capital letters indicate significant differences at *P* <0.05.

Finally, we examined the involvement of GPR173, ERK1/2, and PKA, which are important mediators of intracellular signaling in luteal cells [31, 32] on the effect of PNX-14 on steroid and prostaglandin secretion. All four of the tested siRNA sequences downregulated GPR173 mRNA (Figure 6A, *P* < 0.05) and protein levels (Figure 6B, *P* < 0.05). The siRNA sequences targeting GPR173 in in vitro cultures of luteal cells blocked the stimulatory effect of PNX-14 on steroid and prostaglandin secretion (Figure 6C–F, *P* < 0.05). PD98059, which blocks ERK1/2, abolished the effect of PNX-14 on P_4_, E_2_, and PGF_2α_ secretion but had no effect on PGE_2_ levels (Figure 6C–F, *P* < 0.05). In contrast, pharmacological inhibition of PKA by KT5720 did not alter the PNX-14 effect on steroid secretion (Figure 6C and D, *P* < 0.05).

## Discussion

The decade since the discovery of the neuropeptide PNX has provided an abundance of evidence regarding its regulation of reproductive functions in males and females. Hence, PNX is considered a reproductive hormone [12]. Nevertheless, there is still uncertainty regarding the role of PNX in the regulation of ovarian function in mammals because most of the available evidence relates to the central regulation of the reproductive system by PNX. In addition, most of these findings are based on research conducted in a fish model [15]. Therefore, we investigated the expression and role of PNX-14 in the porcine CL. Our findings indicate that SMIM20/PNX-14 and its receptor GPR173 are expressed in the porcine CL and that their levels change during the estrous cycle. Moreover, we found that PNX-14 regulates the endocrine function of porcine luteal cells by affecting the synthesis of steroids and prostaglandins via GPR173 and ERK1/2.

In the literature, PNX expression has been demonstrated in the ovary of rodents [7, 9], chickens [33], and humans [7], while GPR173 has been detected in rodents [7, 9, 13, 34], cattle [35], and humans [7, 36]. The PNX/GPR173 system has also been described extensively in fish [8, 10, 37]. More precisely, in both human and mouse ovaries, PNX/GPR173 signals have been detected in Gc and theca cells (Tc), and prominently in oocytes. In addition, their expression increases as follicles grow, reaching the highest signal in the secondary and antral ovarian follicles, suggesting the involvement of PNX in folliculogenesis and oocyte maturation, as has already been proved by Nguyen et al. [7]. What is important in the context of our study, besides ovarian follicles, is the strong expression of PNX/GPR173 in human and mouse luteal cells, implying that PNX could play a crucial role in luteal cell physiology [7]. Therefore, we determined the levels of PNX and GPR173 throughout the entire luteal phase—that is, during the formation, full function, and regression of CL. As the results show, expression of *SMIM20*, as well as the PNX-14 protein, increased in CL during the luteal phase. In the case of GPR173, mRNA levels decreased but, conversely, the amount of protein increased. It is well known that post-transcriptional and post-translational modifications often lead to differences between mRNA and protein levels [38]. Differences in GPR173 mRNA and protein expression could also be results of saturation of the receptor under the influence of excess ligand [39]. Such a situation may occur at the mRNA level when we observe a decrease in GPR173 abundance with a simultaneous increase in the expression of the PNX-14 precursor, the SMIM20 protein. As we have shown in the current study, the PNX-14 only in lower doses (1 and 10 nM) can increase the GPR173 protein abundance in luteal cells, which consequently improves PNX signaling. The increasing expression of PNX-14 and GPR173 during the luteal phase suggests that they may support the functioning of the CL, but also play an important role in the physiological regression of the CL, enabling progression of the ovarian cycle.

We investigated the cellular distribution pattern of both proteins and detected PNX-14 in the cytoplasm and GPR173 in the cell membrane mostly in large luteal cells, which are predominantly responsible for the secretion of P_4_ by CL [40]. This observation supports the concept that PNX-14 can regulate the endocrine function of porcine luteal cells. Signals for both PNX-14 and GPR173 have been detected in oocytes, Gc, and Tc of mouse, rats, fish, and humans [7, 9, 10]. In addition, our unpublished data in a porcine model indicate that PNX-14 and GPR173 are expressed in oocytes as well as the Gc and Tc of antral ovarian follicles, which give rise to luteal cells. In plasma, PNX-14 reaches the highest concentration at the beginning of the luteal phase, then decreases slightly. PNX can be produced in many tissues like the heart, pancreas, thymus, stomach, liver, and kidney and is simultaneously transported via blood circulation [4, 6]. Indeed, we observed differences between the local expression of PNX in the CL and the PNX levels circulating in the blood. In pigs, the PNX concentration was around 1 ng/mL, which is higher than what has been detected in the serum of healthy women (0.289 ± 0.05 ng/mL) and in the serum of women with PCOS (0.515 ± 0.04 ng/mL) [16]. Altered levels of PNX and GPR173 are also characteristic for other pathologies of the reproductive system. Women with endometriosis show reduced serum PNX levels as well as a reduced expression of GPR173 in the endometrium [17]. Similarly, in canine cystic endometrial hyperplasia and pyometra, both PNX and GPR173 are downregulated [18]. Thus, insufficient or excessive expression of PNX/GPR173 may be the cause of reproductive disorders.

Hormones produced by luteal cells and transported to the CL, including insulin, LH, P_4_, and prostaglandins, create a unique environment for the proper function of the CL [41]. Therefore, these factors may directly affect the levels of SMIM20/PNX-14 and GPR173, which are locally produced in porcine luteal cells. Interestingly, the results of our study documented that all of the abovementioned factors caused a decrease in the expression of *SMIM20* as well as the secretion of PNX by luteal cells. The results of LH and P_4_ regulation of PNX levels may also partly explain the observed pattern of SMIM20/PNX-14 expression in the CL during the estrous cycle because the LH peak at the beginning of the luteal phase as well as P_4_ levels during the mid-luteal phase may cause lower expression of this peptide in the CL. The available literature does not provide many examples of hormonal regulation of PNX levels. However, in women with PCOS, Ullah et al. [16] observed a positive correlation between the levels of PNX-14 and LH, FSH, total testosterone, and P_4_, and a negative correlation between the levels of PNX-14 and E_2_ and insulin. Similar to our findings, Rybska et al. [18] found that the plasma PNX concentration is negatively correlated with P_4_ in healthy bitches. On the other hand, in cultures of immortalized hypothalamic neurons, estrogen and leptin do not alter the expression of *Smim20* [42]. GPR173 is also regulated by hormones—for example, in the rat hypothalamus, treatment with E_2_ increases its expression [34]. More is known about the effect of PNX on the tested hormones in other tissues. PNX stimulates insulin secretion by pancreatic cells [43] as well as LH release by the pituitary gland [4]. Therefore, there could also be negative feedback between the tested factors and PNX, resulting in a decrease in PNX, to prevent excessive synthesis and disturbance of the physiological response in the CL.

The main function of the CL is the secretion of hormones, in particular P_4_, which is responsible for preparing the uterus for embryo implantation, and in the case of pregnancy, it prevents miscarriage. Most dysfunction of the CL is caused by abnormal, insufficient secretion of P_4_ [44]. Luteal cells also secrete E_2_, which is important because E_2_ metabolites influence angiogenesis in the CL and LH-mediated events [45]. In our in vitro study, PNX-14 significantly increased P_4_ and E_2_ secretion by luteal cells. Furthermore, PNX-14 upregulated gene and protein expression of STAR, CYP11A1, HSD3B, and CYP19A1, the main proteins and enzymes involved in the synthesis of those steroids. These results are consistent with most of the experimental data regarding ovarian steroidogenesis. For example, PNX-20 increases the E_2_ level as well as CYP19A1, LH receptor (LHR), and FSH receptor (FSHR) expression in the human HGrC1 line [7]. In zebrafish, PNX-20 stimulates steroidogenesis: it increases the mRNA expression of many steroidogenic factors, including *cyp11a1*, *cyp17a1*, *cyp19a1*, and *17bhsd*, as well as *er2a* and *er2b* in the ovary [10]. In contrast, Breton et al. [37] performed transcriptome and steroid profiles in female green-spotted puffer (*Dichotomyctere nigroviridis*) and found that steroidogenic enzymes are largely unaffected by PNX-14 treatment. Although they found that PNX-14 administration increased the blood P_4_ levels in females, these changes were not significant. Interestingly, the combined treatment of PNX-14 with LH resulted in a decrease in both the secretion of P_4_ and the expression of steroidogenic proteins involved in its synthesis pathway, compared with the stimulating effect of LH alone. Moreover, PNX-14 decreased protein expression of LHCGR. This observation may partly explain why the combined treatment of LH with PNX-14 results in a decrease in steroid synthesis by luteal cells in comparison to the stimulating effects of LH alone and PNX-14 alone. Thus, we did not observe an additive effect of those two hormones, perhaps due to their mutual negative regulation downregulation in the CL.

Prostaglandins regulate CL formation and regression in pigs, humans, and many other species. PGE_2_ acts at the beginning of the luteal phase, promoting the formation of the CL, while PGF_2a_ has the opposite effect: an increase in its level causes autophagy and apoptosis of luteal cells and, consequently, CL regression [20]. Our data provide evidence of the luteotropic effect of PNX by stimulating PGE_2_ secretion while simultaneously reducing PGF_2a_ levels. We next examined the expression of receptors for these prostaglandins, PTGFR and PTGER2. PGE_2_ may act through the PTGER receptor isoforms, of which PTGER2 is mainly expressed in the porcine CL [46]. Interestingly, PNX-14 downregulated PTGER2 and PTGFR gene and protein expression. The results obtained by us clearly indicate that PNX-14 supports the endocrine function of CL by stimulating P_4_, E_2_, and PGE_2_ secretion by luteal cells and maintains their activity in the middle luteal phase; therefore, it can also be classified as luteotropic factors. Studies on other neuropeptides indicate that they can modulate prostaglandin synthesis. In human luteal cells, ghrelin exerts luteolytic action by reducing PGE_2_ and increasing PGF_2α_ luteal release [47]. In turn, in porcine endometrium orexin affects the mRNA levels of a protein involved in de novo synthesis of prostaglandins, and its effect is dependent on doses, days of the estrous cycle, and periods of pregnancy [48]. It is worth adding that PNX-14 could enhance prostaglandin production because it contains amino acid sequences present in the structure of MITRAC7, a protein controlling the mitochondrial respiratory chain. In addition, the MITRAC complex consists of COX1, which participate in the synthesis of prostaglandins [49, 50].

So far, the initial simulations by Stain et al. [13] on the predicted receptor for PNX peptides have been confirmed. The literature has provided an abundance of evidence that PNX acts in various tissues by interacting with GPR173 and promotes osteoblastic differentiation of MC3T3-E1 cells [51], GnRH neuronal migration [52], and LH release [13]. Our results agree with the study by Nguyen et al. [7], who observed that in human Gc cells, the effect on the expression of steroidogenic factors is suppressed by GPR173 siRNA. We observed that knockout of GPR173 in luteal cells impairs the action of PNX-14 on steroid and prostaglandin secretion, indicating that PNX-14 exerts an effect in the porcine CL through this receptor. In addition, we noted that PNX-14 could trigger several signaling pathways by altering the phosphorylation of kinases. In porcine luteal cells, PNX-14 increased the phosphorylation of ERK1/2 and AKT, decreased the phosphorylation of PKA, and had varied effect on AMPKα and PKC. In rat pancreatic cells, the stimulatory effect of PNX on insulin secretion depends on cyclic AMP (cAMP) signal transduction, while the effect of cell growth and insulin mRNA expression is mediated via ERK1/2 and AKT pathway [43]. Moreover, the cardioprotective effect of PNX in rats is associated with an increase in ERK1/2, AKT, and endothelial nitric oxide synthase (eNOS) phosphorylation [53]. PNX can act through different intracellular pathways; however, we decided to check the possible involvement of ERK1/2 and PKA on the secretory function of luteal cells due to the fact that those kinases are primarily involved in steroid and prostaglandin secretion in luteal cells [24, 31, 32]. Based on the reduction in PKA phosphorylation, we conclude that this signaling pathway does not mediate the effect PNX in luteal cells. Interestingly, data collected regarding the role of PNX-14 in reproductive function show that it exerts its effect through cAMP/PKA signaling in neurons as well as ovarian follicles. A potential explanation can be found in the effect of PNX-14 on LH signaling in the CL. Activation of LHCGR has been shown to trigger cAMP/PKA signaling and is one of the most important pathways in steroid synthesis in both the ovarian follicle and the CL [21]. The decreased activation of PKA by PNX-14 observed in our study could be due to the negative regulation of the LH receptor expression by PNX-14. Moreover, considering that PKC has been shown to be a negative regulator of steroid secretion in porcine and ovine CL [54, 55], decreasing its activation in the long term may also be a mechanism by which PNX-14 stimulates secretion of steroids and exert a luteotropic effect in the porcine CL.

In conclusion, our study clearly indicates that PNX-14 and GPR173 are expressed in porcine luteal cells. Moreover, the PNX level is regulated by several hormones including insulin, LH, P_4_, and prostaglandins. The proper synthesis of steroids and prostaglandins by luteal cells is a crucial element in the functioning of the CL. Our research indicates that PNX-14/GPR173 is a new luteotropic factor in porcine luteal cells due to the fact that it exerts stimulatory effect on steroids and PGE_2_ synthesis and downregulating PGF_2α_ secretion via GPR173 and ERK1/2. The main limitation of our study is that the experiments were performed in vitro, so in the future, luteotropic effect of PNX-14 in porcine CL should be confirmed in in vivo experiments. This would provide an answer to whether the PNX used in livestock farms could help to regulate reproduction in these animals and to support the treatment of luteal phase deficiencies.

## Supplementary Material

Suplementary_Figure_1_ioad138

## Data Availability

The data underlying this article will be shared on reasonable request to the corresponding author.
