# Supplementary material for: Phoenixin-14 as a novel direct regulator of porcine luteal cell functions
Source: Biol Reprod. 2023 Oct 10;110(1):154–68. doi: 10.1093/biolre/ioad138 (PMC10790343; doi:10.1093/biolre/ioad138)
Supplement: Suplementary_Figure_1_ioad138 [file suplementary_figure_1_ioad138.pdf]

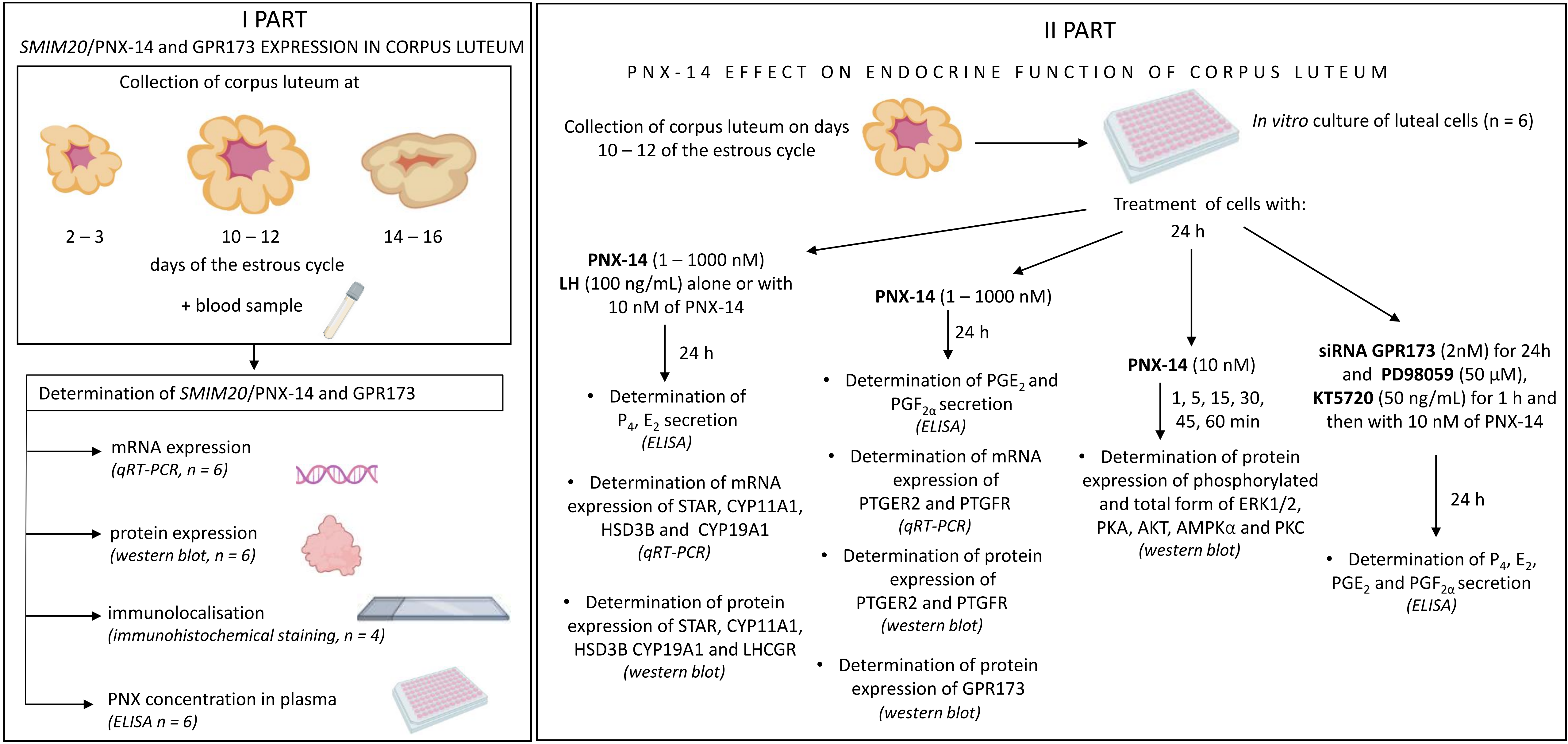

Supplementary Figure 1. Scheme showing the following experiments in the current study. In the first part, we determined *SMM20*/PNX-14 and GPR173 levels in the corpus luteum and the plasma concentration of PNX during the estrous cycle in pigs. In the second part, we conducted a series of *in vitro* cultures of luteal cells from corpus luteum collected on days 10 - 12 of the estrous cycle to examine the effect of PNX-14 on progesterone (P_4), estradiol (E_2), prostaglandin E_2 (PGE_2) and $F_{2\alpha}$ ($PGF_{2\alpha}$) secretion, as well as mRNA and protein expression of steroidogenic factors (STAR, CYP11A1, HSD3B, CYP19A1 and LHCGR) and prostaglandin receptors (PTGER2, PTGFR). Next, we checked the effect of PNX-14 on the activation of extracellular signal-regulated kinases 1/2 (ERK1/2), protein kinase A (PKA), protein kinase B (AKT), 5'AMP-activated protein kinase alpha (AMPKα) and protein kinase C (PKC). Finally, we analyzed the involvement of the GPR173 receptor and ERK1/2 and PKA kinases on PNX-14 effect on steroid and prostaglandin secretion by luteal cells using siRNA GPR173 and pharmacological blockers of kinases, PD09859 and KT5720, respectively.
